# Opposite Roles for p38MAPK-Driven Responses and Reactive Oxygen Species in the Persistence and Resolution of Radiation-Induced Genomic Instability

**DOI:** 10.1371/journal.pone.0108234

**Published:** 2014-10-01

**Authors:** Erica Werner, Huichen Wang, Paul W. Doetsch

**Affiliations:** 1 Department of Biochemistry, Emory University School of Medicine, Atlanta, Georgia, United States of America; 2 Department of Radiation Oncology, Winship Cancer Institute, Emory University School of Medicine, Atlanta, Georgia, United States of America; 3 Department of Hematology and Medical Oncology Winship Cancer Institute, Emory University School of Medicine, Atlanta, Georgia, United States of America; University of Newcastle, United Kingdom

## Abstract

We report the functional and temporal relationship between cellular phenotypes such as oxidative stress, p38MAPK-dependent responses and genomic instability persisting in the progeny of cells exposed to sparsely ionizing low-Linear Energy Transfer (LET) radiation such as X-rays or high-charge and high-energy (HZE) particle high-LET radiation such as ^56^Fe ions. We found that exposure to low and high-LET radiation increased reactive oxygen species (ROS) levels as a threshold-like response induced independently of radiation quality and dose. This response was sustained for two weeks, which is the period of time when genomic instability is evidenced by increased micronucleus formation frequency and DNA damage associated foci. Indicators for another persisting response sharing phenotypes with stress-induced senescence, including beta galactosidase induction, increased nuclear size, p38MAPK activation and IL-8 production, were induced in the absence of cell proliferation arrest during the first, but not the second week following exposure to high-LET radiation. This response was driven by a p38MAPK-dependent mechanism and was affected by radiation quality and dose. This stress response and elevation of ROS affected genomic instability by distinct pathways. Through interference with p38MAPK activity, we show that radiation-induced stress phenotypes promote genomic instability. In contrast, exposure to physiologically relevant doses of hydrogen peroxide or increasing endogenous ROS levels with a catalase inhibitor reduced the level of genomic instability. Our results implicate persistently elevated ROS following exposure to radiation as a factor contributing to genome stabilization.

## Introduction

Sparsely ionizing radiation damages the DNA structure directly by introducing base lesions, as well as single and double strand breaks and indirectly by photons generating reactive oxygen species (ROS) [1]. Particle radiation such as the HZE (High atomic number and energy ions) component of galactic cosmic rays, particle radiation therapy beams or natural radionuclides such as uranium and radon, impose damage through a particle track and by energy deposited radial to this track introducing clustered and complex lesions in the DNA more difficult to repair [2], [3], [4]. Structural damage to DNA is of major significance for cell function and triggers short term cellular responses including further ROS generation in the context of a cellular stress response, changes in gene transcription, signal transduction and induction of cell cycle arrest to ensure that most of the damage is repaired within a few hours [5], [6].

While most studies on the biological consequences of radiation have focused on early events leading to DNA repair, cell survival or death upon high dose exposures, less is known on how exposure to moderate doses of radiation induces delayed and persistent phenotypes in the progeny of targeted and adjacent non-targeted cells, leading to alterations in tissue homeostasis, genomic instability and cancer [7] and whether these responses differ among low- and high- LET sources of radiation. These responses occurring within the first days to weeks following exposure have been shown to vary with radiation quality and dose, and may determine the future consequences of the exposure, leading to complete repair, repopulation of a radiation targeted tumor, tissue inflammation amplifying the initial damage, fibrosis or emergence of cancer, depending on the biological setting. Thus, further understanding of the persisting phenotypes present in the fraction of cells surviving the exposure should provide tools to measure individual variations in sensitivity and responses to radiation as well as to predict or modify outcomes following radiation therapy or environmental exposures on earth or during prolonged space flight.

Studies in lung tissue are of significance as this organ is the first target for environmental alpha particle emitters such as uranium and radon, which are responsible for a significant fraction of lung cancer incidence in non-smokers [8]. For astronauts, lung cancer development poses a significant risk for death as a consequence of extended space missions [9]. Additionally, the radiosensitivity of this organ is dose-limiting for radiation therapy and a source for acute and long-term complications in the treatment of multiple thoracic cancers [10], [11].

Genomic instability is one of the persisting phenotypic signatures induced over a wide dose range of radiation and is proposed to drive carcinogenesis [12]. Post-exposure persisting genomic instability has been detected in uranium miners [13] and in astronauts in proportion to their cumulative mission time [14]. Large cohort multi-investigator studies have shown a positive correlation between genomic instability and later cancer development [15]. Genomic instability is defined by the accumulation of multiple changes leading to the conversion of a stable to an unstable genome, characteristic of most tumors. These changes may appear de novo in the progeny of surviving cells and involve large-scale chromosomal rearrangements and aberrations, gene amplification, aneuploidy, micronucleus formation, microsatelite instability and gene mutations [16], [17] and are transmitted to the subsequent progeny [18]. In contrast to low-LET radiation, for which current models suggest a threshold effect, a single alpha particle is sufficient to induce mutations [19]. While doses in the therapy range are clearly more effective than low LET radiation in terms of cell killing, the effects of high LET radiation on other cellular responses remain largely unknown.

ROS have been suggested to play a significant, though still controversial role promoting and sustaining these persistent phenotypes [20]. Elevated ROS levels have been observed for multiple cell doublings in clones and cell lines derived from X- irradiated cells [21], [22], where they have been associated with genomic instability [23], including delayed non-clonal chromosomal aberrations [20], [24], [25], as well as increased mutation rates [23], [26]. Elevated ROS levels persist in non-replicating cells *in* *vitro* and *in* *vivo* in mouse tissue following exposure to radiation [27]. However, while there is a clear association of elevated ROS with genomic instability, it is not known whether both phenotypes are directly related or result from the expression of other persistent phenotypes associated with tissue damage and senescence [28], [29], [30]. Furthermore, while radiation quality does affect genomic instability [31], [32], [33], it is unknown whether the type of damage generated or the elicited biological effects of high-LET radiation influence the ROS response.

To address how elevated ROS, genomic instability and other cellular responses are related, we studied the surviving fraction of irradiated cells, which are the progeny of cells that recovered from the initial ionizing radiation-inflicted damage. Such cells are relevant for revealing long term effects of exposure to radiation, including genomic instability, disrupted homeostasis and transformation [34]. We exposed human bronchial epithelial cells with functional p53, immortalized by telomerase and CDK4 (HBEC-3KT) to moderate doses of low (X-rays) and high (high energy ^56^Fe ions) LET radiation, as they introduce qualitatively and quantitatively different types of DNA damage, and measured ROS levels as well as various indicators of genomic instability.

In this cell type, we found that the radiation-induced ROS response is not determined by the quality of the initial DNA damage and persists over a time period when indicators for genomic instability can be detected. A senescence response driven by p38MAPK is induced in a radiation quality and dose dependent manner, but persists for a shorter time period compared to genomic instability and ROS. We further show that both responses, elevated ROS and p38MAPK-driven phenotypes, have distinct effects and can be manipulated to reduce genomic instability.

## Materials and Methods

### Reagents, cell culture and irradiations

All reagents, unless stated, were obtained from Sigma (St. Louis, MO, USA). HBEC-3KT cells [35], a gift from Michael Story (UT Southwestern), were cultured in Keratinocyte Serum-Free media (Invitrogen, Carlsbad, CA, USA). High-LET irradiation was carried out using an alternating-gradient synchrotron (AGS) ^56^Fe ions, 600 MeV/amu, LET: 174 keV/µm at NASA Space Radiation Research Laboratory at Brookhaven National Laboratory (Upton, NY). Low-LET irradiation was carried out using an X-ray machine (X-RAD320, Precision X-Ray, N. Branford, CT, USA) at 320 kV, 10 mA. The dose rates for high and low-LET radiation were about 1 Gy/min. Every irradiation was carried out on triplicate samples (500,000 cells per T25 flask), which were kept proliferating and passaged independently twice a week.

### H_2_O_2_ treatments

Cells were washed 1x with PBS and then incubated for 15 minutes with H_2_O_2_ diluted in PBS at the indicated concentration using the molar extinction coefficient to estimate the concentration of the commercial stock solution (Sigma).

### RNA interference

On-target siRNA SMART pool oligonucleotides (Thermo Scientific, Waltham, MA, USA) directed to p38α or ATM and a scrambled sequence were double transfected with Lipofectamine 2000 (Invitrogen) starting the 3rd day following irradiation with one day of recovery between transfections. Cells were analyzed two days after the second transfection.

### ROS measurements by flow cytometry

Cells were incubated in suspension with 4 µg/ml 2,7-Dichlorodihydrofluorescein diacetate or 3 µg/ml Dihydroethidium (Invitrogen) in PBS or attached to the plate with 1 µg/ml MitoSox (Invitrogen) in media for 30 min at 37°C. Fluorescence of 10,000 cells was acquired in a LSRII Cytometer (BD, Franklin Lakes, NY, USA) and analyzed using FlowJo (TreeStar Inc. Ashland, OR, USA). Each determination was performed in triplicate wells. To calculate the fold of induction, the average of the readings in non-irradiated replicates was defined as 1.

### Cytokinesis-block micronucleus assay

Cells were plated at 20,000/well on glass coverslips. Following treatments, cells were incubated for 18 h with 3 µg/ml Cytochalasin B in media [36], fixed in 4% paraformaldehyde and stained with 4′,6-Diamidino-2-phenylindoledihydrochloride (DAPI). 200 or more binucleated cells were counted as needed to score 10 micronucleus/nuclear bud per sample, two replicates per condition. Nuclear buds were included as this is a frequent aberration induced by radiation and may reflect micronucleus precursors [37] or gene amplifications [38], [39].

### Immunofluorescence microscopy

Cells were fixed in 4% paraformaldehyde and permeabilized with 0.2% TritonX-100. Antibodies used were γH2AX (Millipore, Billerica, MA, USA), 53BP1 (Novus Biologicals, Littleton, CO, USA), BrdU, IL-8 (BD Biosciences, San Jose CA, USA), phospho-p38MAPK (Cell Signaling, Danvers, MA, USA). Proliferation was measured by incubating the cells for 8 h with 10 µM BrdU, washed with fresh media for 30 min, fixed and stained using an antigen retrieval step before blocking. For intracellular IL-8 staining, cells were treated with 10 µg/ml Brefeldin A or 2 µM Monensin for 4 h prior fixation. Then they were fixed and permeabilized using saponin. Cells were imaged using a Olympus IX81 inverted microscope with QCapture (QImage, Surrey, CAN), while cells stained for IRIF were imaged in a Zeiss LSM510META confocal microscope (Thornwood, NY, USA) using a 20×Plan-Apo objective (NA = 0.75). Images were processed using contrast/brightness enhancement only. The percentage of proliferation was determined by counting and averaging the cells of 5 independent fields for each sample. IRIF were counted in at least 5 different fields totaling 50 cells in duplicate irradiations.

### Senescence associated beta-galactosidase activity

As published [40], cells were incubated for 16 h at 37°C for the precipitate to accumulate, washed and photographed. While this technique distinguished senescence induced by 10Gy X-rays in human primary fibroblasts (Figure S2a), HBEC-3KT cells had a higher background activity, thus X-gal staining in 50 cells was quantified measuring the raw pixel intensity per cell after background subtraction using ImageJ (National Institute of Health, USA).

### Cytokine Elisa

50,000 cells were plated in triplicate and incubated for 24 h in media without supplements but with 0.02%BSA. IL-8 or IL-6 were measured by Elisa (R&D Systems, Minneapolis, MN, USA). Measurements were corrected using crystal violet staining of the cells remaining in the well after supernatant collection.

### Statistical analysis

The error bar in the figures represents the standard deviation when associated to the average of independent samples irradiated simultaneously or the standard error when associated to the average of multiple independent irradiations. Excel was used for paired two-tailed Student t-test assuming equal variance of the samples. Graph Pad Software was used for analysis of variance as indicated in the figure legends. Vassarstats was used to compare the frequency distributions of the number of foci per cells using a chi-squared test.

## Results

### Elevated ROS levels persist and are independent of radiation quality

**Figure 1:**
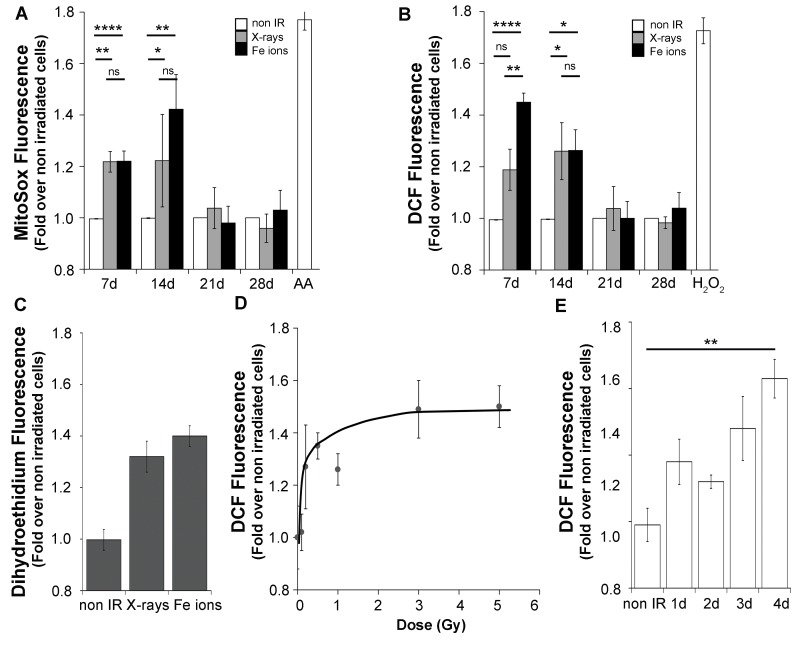
ROS response is independent of radiation quality and persists for 14 days. Flow cytometry detecting multiple ROS species at day seven following exposure to 1Gy X-rays or Fe ions. Time course of MitoSox (a) or DCF (b) fluorescence induced by 1Gy of radiation. As controls, 2 µg/ml Antimycin A was added during the loading period to non-irradiated cells or non-irradiated cells loaded with H_2_DCFDA were resuspended in 5 µM H_2_O_2_. The values represent the averages of duplicate determinations over 3 independent irradiations. Error bars represent SEM. One-way ANOVA with Bonferroni comparison of all columns *p<0.05, **p<0.005, ***p<0.0001. (c) Dihydroethidium fluorescence at day 7 post-irradiation. Error bars represent SD. (d) ROS levels seven days following exposure to the indicated dose of X-rays. Error bars represent SD. (e) DCF levels measured at the indicated time points following exposure to 3Gy X-rays. Error bars represent SD. Paired Student's t-test **p<0.005.

To examine the effects of the initial burden and quality of DNA damage on ROS production, we employed a 1Gy dose, which is sufficient to damage the majority of the irradiated cells in the cultures by X-ray photons (low-LET) or by at least one Fe ion (high-LET) track [41], [42]. This is a dose established for gamma radiation to increase cancer risk in humans causing a relatively small fraction of 3KT cell inactivation (10%, Figure S1c) and sufficient for particle radiation to induce cytogenetic changes detectable *in* *vivo* [43], [44], and well below doses used to induce cell senescence. Measurements were made at day 7, when cultures have undergone about 2 population doublings (40 h doubling time) following exposure to both radiation types. One week following exposure, we detected increased ROS levels induced by both types of radiation using multiple probes, detecting mitochondrial superoxide (MitoSox, Figure 1a), a general oxidant sensitive fluorescent probe (2,7 dichlorodihydrofluorescein diacetate, which detects a variety of ROS species [45], Figure 1b) and total cellular superoxide (dihydroethidium, Figure 1c), Measurements of MitoSox (Figure 1a) or DCF (Figure 1b) revealed that the ROS response is sustained for 2 weeks or over 8 population doublings in response to both types of radiation. Furthermore, sustained ROS was detected in another human bronchial epithelial cell line (NL20) and in BJ fibroblasts (not shown), indicating that this is a general response to ionizing radiation exposure. While the increase in fluorescence average intensity of the whole population was relatively small, the whole cell population shifted toward higher values (Figure S1a) and occurred in cells in G1 as well as in G2 phases of the cell cycle (Figure S1b). Although exposure to Fe ions appeared to induce a more robust response, the difference compared to X-rays was not statistically different under these experimental conditions. These results show that ROS levels increase in response to radiation induced DNA damage in the majority of the cells in the cultures, and suggest that the response is independent of the amount and complexity of the DNA lesions caused by the exposure to radiation of distinct quality. We further tested this concept by measuring ROS levels as a function of X-ray dose, which at day 7 post-exposure displayed a threshold-like response, where a signal was detected for doses above 0.2Gy and approached a plateau level by 1Gy, sustained through 3Gy (Figure 1d). ROS measurements at earlier times following exposure to 3Gy X-rays revealed that this increase can be detected from the second day following irradiation (Figure 1e). Thus, we examined next whether elevated ROS are associated with other persistent responses triggered by radiation.

#### ROS response overlaps in time with radiation-induced persistent genomic instability

**Figure 2:**
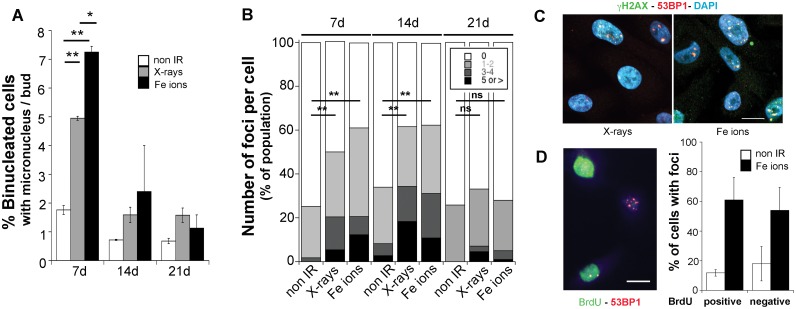
Exposure to radiation induces persistent genomic instability. (a) Time course of micronucleus frequency in binucleated cells exposed to 1Gy X-rays or Fe ions (average of two independent irradiations, error bars represent SEM). Paired Student's t-test, *p<0.05, **p<0.01. (b) Time course of γH2AX- 53BP1 positive foci per cell distribution in a population of 50 cells per condition following exposure to 1Gy of the indicated radiation. One of two experiments is shown. Chi-Square test NI vs. X-rays or Fe ions. day 7 and day 14 p<0.001. Day 21 p>0.05. (c) Immunofluorescence staining of γH2AX-53BP1 positive foci 7 days following exposure to 1Gy of the indicated radiation type. Scale bar is 10 µm. (d) Immunofluorescence staining for BrdU and 53BP1 7 days following exposure to 1Gy Fe ions. Scale bar is 10 µm. The graph represents the average of scoring in 5 different fields a total of 70 cells.

To address the relationship of ROS levels with genomic instability persisting following repair of the initial lesions, a response directly related to radiation quality [32], we evaluated two biological endpoints: i) the frequency of micronucleus formation as a reflection of gross structural chromosomal abnormalities and ii) persistent DNA damage evidenced by immunofluorescence detection of phosphorylated H2AX (γH2AX) and 53BP1, recruited to chromatin proximal to a double strand break (DSB), where they co-localize in ionizing radiation-induced foci (IRIF) for DNA damage sensing and repair. The cytokinesis-block micronucleus assay detects DSB misrepair leading to asymmetrical chromatid exchanges as well as chromosome fragments, which are missegregated during mitosis and become excluded from the nucleus into a smaller extranuclear body (micronucleus) detected at the interphase following mitosis and scored in binucleated cells [36]. This assay is highly sensitive and has been used for *in* *vivo* biodosimetry to measure exposure to low and high-LET radiation [44]. We detected persistent micronuclei formation at day 7, with frequencies increasing from 2.48% (+/−0.9 SEM) in non-irradiated to 4.7% (+/−1.2 SEM) following X-rays (1.9-fold increase) and to 11.9% (+/−4.5 SEM) following Fe ions (4.8-fold increase) exposure. In addition to exhibiting a robust radiation quality effect, this phenotype was transient, persisting through day 14, when approximately a 2-fold and a 3-fold increase over non-irradiated cells was detected for X-rays and Fe ions, respectively, By day 21, micronucleus formation frequencies were not different from non-irradiated cells (Figure 2a).

At early times following acute exposure to ionizing radiation, the number of IRIF reflects the number of DSB measured by physical methods [46]. Seven days following exposure to radiation, we detected persistent γH2AX-53BP1 positive foci in a large fraction of the cell population, 55.45% (+/−5.8 SEM) and 66.6% (+/−5.3 SEM) of cells previously exposed to X-rays or Fe ions, respectively, compared to 20.7% (+/−5.07 SEM) of non-irradiated cells. Cells exposed to Fe ions displayed large IRIF (1 µm in diameter or larger) and higher frequencies per cell (Figure 2 b, c). Increased IRIF frequency was still detected at day 14 following exposure, but similar to the temporal course of micronucleus occurrence, at day 21, the number of cells with such IRIF was not statistically different under all three conditions. Thus, while the presence of residual IRIF was common for both types of radiation, which indicates persistent activation of the DNA damage response (DDR), cells exposed to Fe ions exhibited a larger population with multiple IRIF suggesting higher levels of residual damage in cells exposed to high-LET radiation compared to low-LET radiation.

To examine whether the presence of IRIF is restricted to a small sub-population of highly damaged and non-proliferating cells, we monitored the presence of 53BP1-positive IRIF in cultures labeled with BrdU. Cells were pulsed at day 7 for 8 h with BrdU and analyzed for 53BP1 IRIF and BrdU incorporation by immunofluorescence. The percentage of cells labeled with BrdU in the different groups was not significantly different (57.3% +/−6.7 SEM for non-irradiated cells vs. 53.85% +/−6 SEM, p>0.5 for Fe ions), which indicates a normal proliferation rate. We found similar number of cells positive for 53BP1 foci in cells that stained negative as well as positive for BrdU (Figure 2d), indicating that foci are present in actively proliferating cells and likely at multiple stages of the cell cycle. Collectively, these results indicate that genomic instability, measured by two different endpoints, displays radiation quality dependence and persists for 2 weeks in a proliferating fraction of cells following exposure to radiation. Importantly, because of distinct quality and dose response profiles, these results exclude elevated ROS as a causal factor for persistent genomic instability in this system.

#### Expression of senescence-associated phenotypes depends on radiation quality

**Figure 3:**
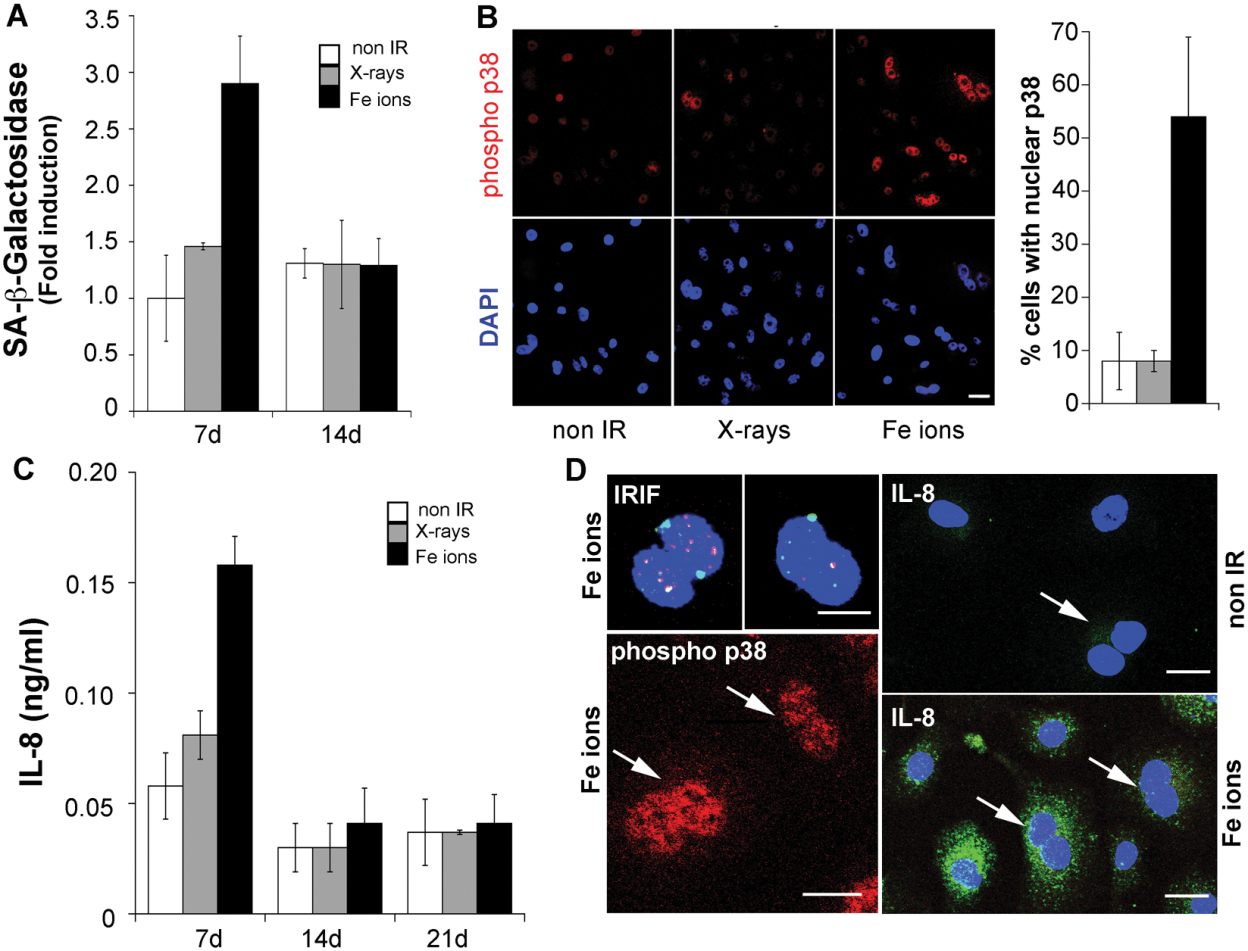
Exposure to radiation induces transient phenotypes shared with senescence. (a) Time course of senescence-associated beta galactosidase activity following exposure to 1Gy X-rays or Fe ions. (b) Immunofluorescence staining for phosphorylated p38MAPK seven days following exposure to 1Gy X-rays or Fe ions. The graph represents the average of 100 cells scored in 4 different fields. (c) Time course of IL-8 production by cells following exposure to 1Gy X-rays or Fe ions. (d) Immunofluorescence for the indicated markers in cells at day 7 post irradiation, incubated with 3 µg/ml cytochalasin B for 6 h prior fixation. For intracellular IL-8 detection, the cells were incubated with monensin as described in Figure S2 legend. Arrows point to binucleated cells. Scale bars = 20 µm. All panels show one representative experiment of two. All error bars represent SD.

Elevated ROS levels and persistent IRIF have been previously reported to be associated with radiation-induced senescence. Exposure to a high dose (10Gy) of X-rays induces senescence in human fibroblasts one week following exposure (Figure S2a, [47]) and this response is characterized by p38MAPK activity, growth arrest, increased beta-galactosidase activity and the secretion of cytokines, chemokines and tissue remodeling factors [48]. To examine whether ROS are elevated as a consequence of senescence, we tested next whether exposure to 1Gy X-rays or Fe ions induces changes also associated with senescence in HBEC-3KT cells. We found that exposure to Fe ions, but not X-rays, caused a 3-fold increase in the average senescence-associated beta-galactosidase activity per cell detected at day 7, but not at day 14 (Figure 3a). While it is unknown how beta galactosidase activity increases are related to senescence and it's activity might increase as a consequence of other processes occurring in the cell [49], [50], this phenotype was accompanied by an increase in nuclear size (Figure S2b). Exposure to Fe ions, also induced phosphorylated p38MAPK translocation to the nucleus in a significant fraction (59.2% +/−6.5 SD) of the cell population compared to 12.4%(+/−6.5 SD) X-ray irradiated or 13.9% (+/−10.7 SD) non-irradiated cells (Figure 3b). No nuclear total or phosphorylated p38MAPK was detected in non irradiated cells (Figure S3c). In addition, at this dose, only exposure to high-LET radiation induced the production of IL-8, detected in the media at day 7, but not at day 14 following exposure (Figure 3c). In contrast, IL-6, another cytokine secreted by fibroblasts during senescence, was not detected (not shown). Similar to IRIF, we detected nuclear phospho-p38 and IL-8 production by immunofluorescence in proliferating cells as evidenced by positive signals in binucleated cells in cultures incubated with Cytochalasin B (Figure 3d). Given the clear effect of radiation quality on the induction of these phenotypes, we tested whether IL-8 could be induced by higher doses of low-LET radiation. HBEC-3KT cells exposed to increasing X-rays doses, released IL-8 into the media, detectable at day 7 at 3–4Gy and increased exponentially at higher dose (Figure S3a), correlating with reduced clonogenic growth potential (Figure S1c). Similarly, 7 days following an exposure to 6Gy X-rays, we detected IL-8 production, 53BP1 foci and nuclear phospho p38 in proliferating cells, evidenced by signal positive binucleated cells after Cytochalasin B treatment (Figure S3b). These results indicate that high-LET radiation is much more effective in inducing these phenotypes shared with senescence at low dose, but this response can not be defined as senescence as it is transient and arises in the context of proliferating cells. The fact that IL-8 can also be induced by low-LET radiation at high doses, suggests that these phenotypes are induced in response to the quality of the initial DNA damage.

#### The senescence-associated phenotype is driven by p38MAPK, promotes genomic instability and limits ROS levels

**Figure 4:**
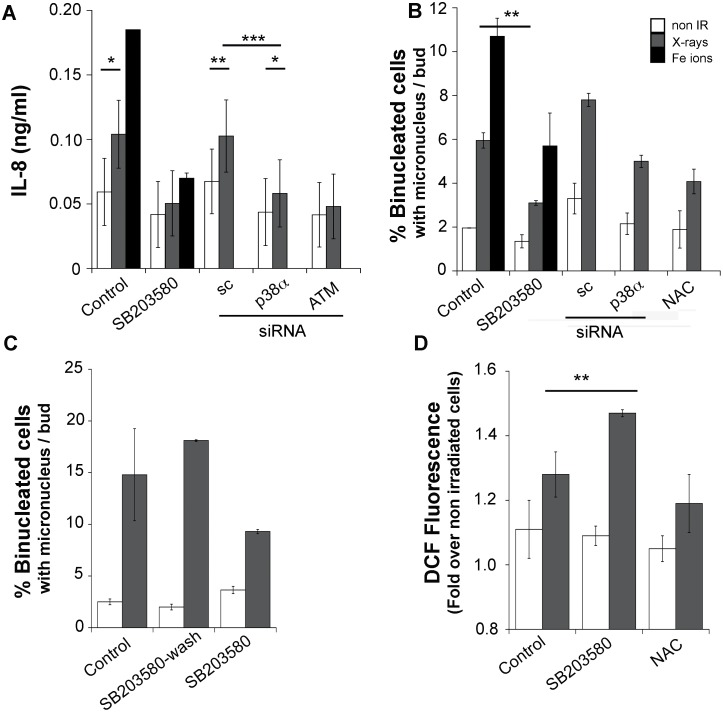
The p38MAPK driven phenotypes promote genomic instability. (a) ATM or p38MAPK inhibition with the chemical inhibitors SB200358 (10 µM) or by RNA interference reduce IL-8 production 7 days following exposure to 3Gy X-rays or 1Gy Fe ions. Inhibitors were added at the moment of incubating the cells to collect conditioned media. Student's t test *p<0.05. Two-way ANOVA, *p<0.05 **p<0.01 ***p<0.005 (b) Interference with p38MAPK activity using an inhibitor or siRNA, as well as 1 mM NAC treatment (control), reduces micronucleus frequency at day 7 following exposure to 3Gy X-rays or 1Gy Fe ions. Two-way ANOVA, **p<0.01. (c) Inhibitory effect of SB200358 is transient: at day 6 post-irradiation with 3Gy X-rays, cells were treated with 10 µM SB 200358 for 18 h and washed in media without inhibitor for 12 h. Micronucleus assay was then performed as indicated in materials and methods. (d) Seven days following exposure to 3Gy X-rays, p38MAPK was inhibited for 8 h and ROS levels were measured by flow cytometry using H_2_DCFDA. Two-way ANOVA, **p<0.01. All panels show one representative experiment of two. All error bars represent SD.

Because some human progeroid syndromes are linked to genomic instability, p38MAPK activation and altered ROS levels [51], [52], we addressed next whether the radiation induced phenotypes shared with senescence play a role in sustaining elevated ROS and/or increased micronucleus frequencies. We performed further studies examining the response to a 3Gy dose of X-rays which induced IL-8, ROS and increased micronucleus formation frequencies to levels similar to 1Gy Fe ions (see below). We disrupted p38MAPK activity with a well-established, selective chemical inhibitor, SB203580 [53] or by interfering with expression using siRNA transfection (Figure S4). The p38MAPK inhibitor reduced IL-8 production induced by both radiation types when added 24 h prior to cell supernatant collection (Figure 4a). Inhibition was observed with siRNA transfection targeting the expression of p38MAPKα or ATM kinases indicating that this phenotype is driven by the activity of both kinases, an observation similar to previously published studies employing high doses of low-LET radiation [48], [54], [55].

Since our results and a previous report [48] suggested that interfering with p38MAPK activity is sufficient to abrogate the secretory response, we tested next whether p38MAPK activity modifies genomic instability measured by the micronucleus assay. Following exposure to radiation, cells were transfected with siRNA or incubated with the p38MAPK inhibitor during the 18 h Cytochalasin B treatment. As shown in Figure 4b, exposure to 3Gy X-rays or 1Gy Fe ions increased micronucleus frequency, which was reduced by the p38MAPK inhibitor. Interference with p38MAPK function significantly reduced micronucleus frequency in irradiated cells (p<0.05) without affecting the frequency in non-irradiated cells (p>0.5). To exclude the possibility that this effect is the result of p38MAPK inhibition causing the selective death of cells harboring the highest level of genomic instability, which are revealed in the micronucleus assay, we determined next whether the effects of p38MAPK inhibition are reversible. At day 6 following exposure to 3Gy X-rays, the cells were plated and a subgroup treated for 18 h with SB203580 and washed for 8 h prior to the assay. Micronucleus formation frequencies were measured at day 7 and compared to non-treated or SB203580 treated cells (Figure 4c). The result shows that cells previously treated with the p38MAPK inhibitor, recover after washing the inhibitor and display elevated micronucleus frequency phenotype, indicating that inhibition by SB203580 is transient and that increased micronucleus formation is a response sustained by persistent p38MAPK activity.

To evaluate whether p38MAPK contributes to elevated ROS levels, we measured the effect of kinase inhibition on ROS production. While the DCF fluorescence signal was modestly reduced by treatment with 1 mM NAC, it was increased following an 8 h treatment with the p38MAPK inhibitor (Figure 4d). In contrast, the inhibitor did not affect mitochondrial superoxide levels (not shown), suggesting that p38MAPK is regulating cellular redox status without affecting superoxide generation in mitochondria. This result is consistent with radiation induced ROS production and is independent of the phenotypes shared with senescence and that, in contrast, the p38MAPK-driven response downregulates the overall cellular ROS levels. Moreover, this result indicates that moderately elevated levels of ROS may be involved in reducing genomic instability.

#### Increased ROS levels reduce genomic instability

**Figure 5:**
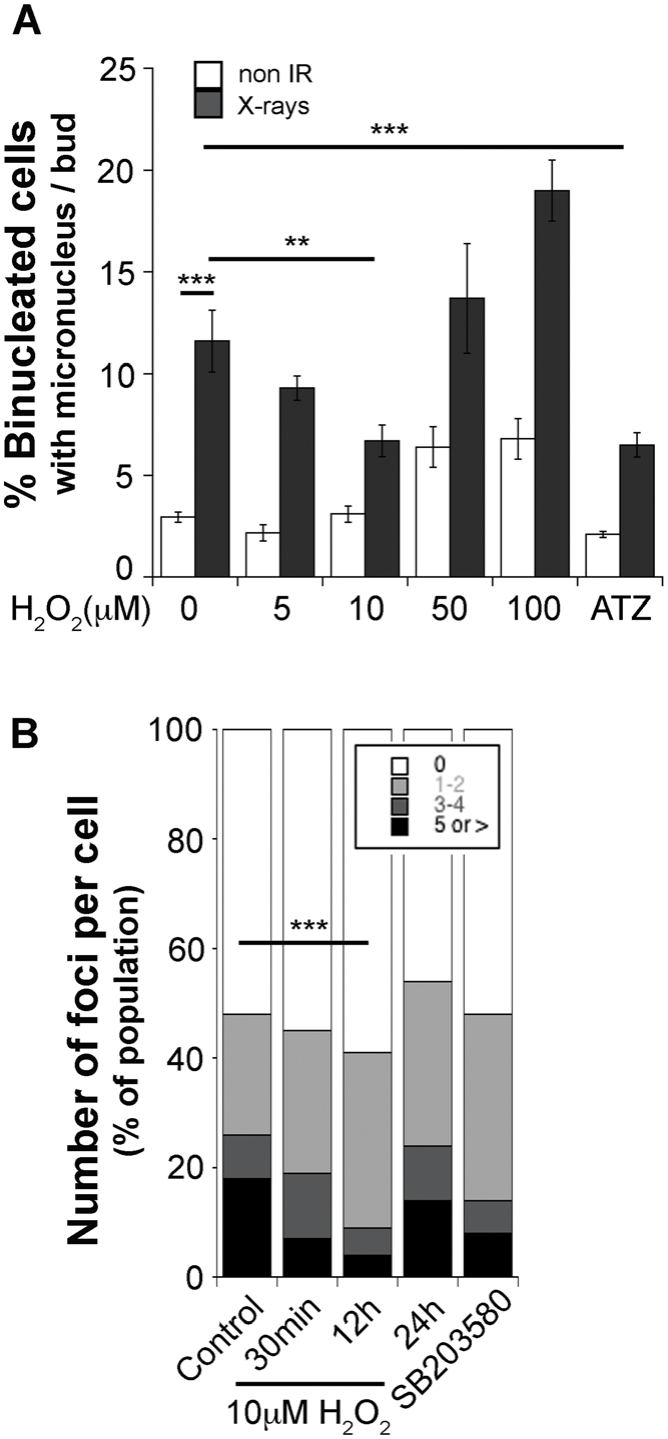
Exposure to low doses of exogenous H_2_O_2_ reduces genomic instability. Non-irradiated and 3Gy X-rays irradiated cells were treated at day seven for 15 minutes with the indicated concentration of H_2_O_2_ in PBS, followed by replacement to normal media (IRIF staining) or supplemented with cytochalasin B (micronucleus assay). (a) Dose dependent effect of exogenous H_2_O_2_ on micronucleus frequency. As a control, cells were co-incubated with 10 mM 3 amino 1,2,4 triazole (ATZ). Error bars represent SEM. ANOVA with Bonferroni correction **p<0.01, ***p<0.005. Non irradiated cells with 10 µM H_2_O_2_ or ATZ and without treatment were not statistically different. (b) γH2AX-53BP1 foci distribution at the indicated time following treatment of irradiated cells with 10 µM H_2_O_2_ or a 24 h p38MAPK inhibitor treatment. Chi-Square test Control vs. 12 h H_2_O_2_ p<0.001. The panel shows one representative experiment of two.

To test whether moderate increases in ROS levels can reduce genomic instability, we increased H_2_O_2_ levels in irradiated and non-irradiated cells with the catalase inhibitor ATZ (3-amino1,2,4-triazole) [56], to promote endogenous H_2_O_2_ accumulation as well as by direct exposure to increasing doses of exogenous H_2_O_2_ for 15 min prior to Cytochalasin B addition. Surprisingly, a brief treatment with low doses of H_2_O_2_ was sufficient to reduce micronucleus formation frequency in day 7 irradiated cells without affecting non-irradiated cells, while doses above 50 µM increased frequencies in both non-irradiated and irradiated cells (Figure 5a). A similar positive effect was observed in ATZ-treated (catalase inhibited) cells. These results support a mechanism where ROS are produced to activate pathways to reduce DNA damage and suggest that ROS will reduce the incidence of IRIF as well. Addition of low doses of H_2_O_2_ to day 7 post-irradiated cells reduced IRIF frequency per cell (Figure 5b). Interestingly, while exogenous H_2_O_2_ treatment does not affect IRIF detection (Figure S5a), it reduced the number of IRIF detected as early as 30 min, peaking at 12 h, and reversing by 24 h post treatment. As expected from our previous results, treatment with the p38MAPK inhibitor reduced IRIF frequencies as well. Collectively, these results support a mechanism where a moderate increase in ROS levels is sufficient to trigger signaling pathways that reduce the burden of persistent DNA damage and contribute to the resolution of genomic instability, while greater levels of ROS promote DNA damage and genomic instability, which is consistent with the observed effects of NAC.

## Discussion

In these studies, we have examined the functional relationships of three responses that reflect the persistent effects of cellular exposure to ionizing radiation: genomic instability, reactive oxygen species and p38MAPK-driven phenotypes, which we propose are part of a stress response.

**Figure 6:**
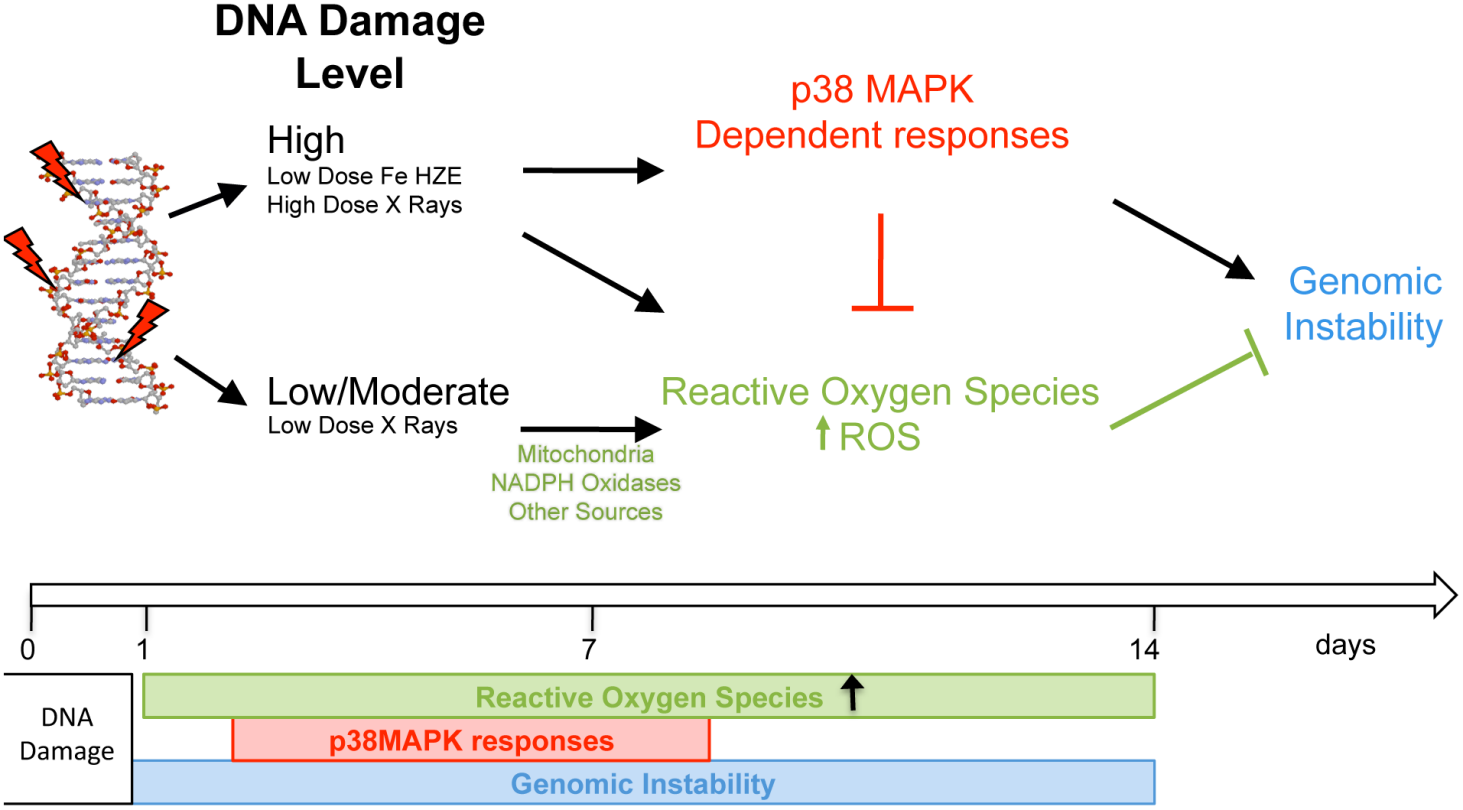
Opposite effects of radiation-induced ROS and p38MAPK driven responses on genomic instability. Summary diagram illustrating the proposed functional and temporal relationships among radiation-induced stress phenotypes, ROS and genomic instability. TOP: p38MAPK driven responses are induced by a mechanism dependent on radiation quality and dose and promotes genomic instability. Both, high and low-LET radiation, induce moderate increases in cellular ROS levels, involved in an operational system that reduces genomic instability. One of the likely mechanisms by which p38MAPK increases genomic instability is through reduction of ROS levels in the cells. BOTTOM: Time course of radiation-induced responses persisting in the surviving cell population.

As illustrated in Figure 6, DNA damage induced by low- and high-LET radiation is sufficient to increase ROS levels, which are sustained for the time that genomic instability persists. Increased DNA damage caused by high-dose low-LET or low-dose high-LET radiation induced the expression of p38MAPK-driven phenotypes of a short duration. By employing selective chemical inhibitors and genetic approaches, we show that this p38MAPK-driven response, exacerbates genomic instability and reduces ROS production. We propose that this effect on the cellular redox mechanism might be involved in promoting genomic instability, as we show that increasing ROS levels by addition of exogenous H_2_O_2_ or catalase inhibition, is sufficient to reduce the expression of genomic instability biomarkers. Thus, contrary to previously suspected roles for promoting DNA damage, our findings point to a positive role for low to moderate levels of ROS in reducing genomic instability.

While previous studies have implicated ROS in increasing and sustaining genomic instability by causing chromosomal damage and increasing mutagenesis [23], [57], our findings reveal a more complex relationship dependent on the range of effective ROS levels. Our studies confirm that radiation induces moderately elevated ROS levels downstream of DNA damage, but surprisingly, are implicated in a homeostatic role rather than driving genomic instability. The ROS levels induced were not directly proportional to radiation dose or quality and were not related to the magnitude of persistent genomic instability induced by each radiation type as would be expected for a causative agent of genomic instability (compare Figures 1a, b with 2a). Consistent with a positive biological role, the ROS levels detected are comparable to those generated by exposure to low doses (between 2 and 5 µM) of exogenously added H_2_O_2_ (Figure S5b), and are within a physiologically relevant range as resting neutrophils release about 0.3 µM H_2_O_2_ and up to 12 µM when activated [58]. Furthermore, we show that exposure to low, but not high doses of exogenous H_2_O_2_, reduces genomic instability in X-ray irradiated cells (Figure 5a and b).

The rapid kinetics of the response induced by exogenous H_2_O_2_ is consistent with a mechanism where a moderately pro-oxidant environment affects DNA repair directly or indirectly via the induction of a transcriptional response. Supporting such a mechanism, low doses of exogenous H_2_O_2_ increase the repair of low dose radiation damage by inducing the expression of the same genes induced by a higher dose of radiation through an adaptive response [59], however it remains to be determined whether ROS triggers cellular hormesis or adaptive pathways similar to low doses of radiation (Recently reviewed by [60]. Our group has previously shown that DNA damage increases cellular ROS [61] and the activation of Yap-1, a transcription factor inducing antioxidant responses and DNA repair enzymes in budding yeast [62]. We speculate that in a long-term or chronic state of genomic instability such as those investigated in these studies, ROS are continuously produced in response to the presence of DNA damage to activate and maintain such pathways. However, their effect on genomic instability will be modulated by the signaling context in which they are produced, including antioxidant levels or p38MAPK-driven response. Consistent with this mechanism, we show that altering the redox status of cells towards increased ROS levels by interference with p38MAPK (Figure 4d) or by inhibiting catalase activity (Figures 5a, b) is sufficient to reduce genomic instability.

Concomitantly with elevated ROS levels, we detected persistent genomic instability in proliferating cells that displayed radiation quality and quantity dependence, consistent with reported findings [63], [64]. Exposure to high-LET radiation induced genomic instability with increased biological effectiveness, which has been attributed predominantly to error prone repair of the complex lesions generated by this type of radiation, which may lead to persistent alterations in DNA structure including amplifications, and duplications [65]. However, our findings indicate that p38MAPK and downstream responses are a separate component contributing to increased genomic instability.

It is not known whether and how both indicators of genomic instability, micronucleus formation and IRIF, are associated. However, our results revealing the transient effect of increasing ROS levels (Figures 5b) and p38MAPK inhibition (Figure 4c) on both indicators, suggest that they respond to a persistent signal, which is being propagated in the progeny of irradiated cells and thus could be associated with the structure of DNA or chromosomes. It remains unclear whether the persistent, residual IRIF represent sites of actual DSB, repair centers where multiple DSB coalesce [64], [66], or disruptions in the chromatin structure [67], [68]. However, in a normal physiological context, the initiating signal recruiting these proteins is a DSB [69], and elevated endogenous γH2AX has been associated with genomic instability in cancer cell lines as well as telomere damage during senescence [70], [71]. Radiation is a known inducer of delayed chromosomal translocations, that would occur through non-programmed DSB formation generated at chromosomal fragile sites, usually rich in repetitive sequences where replication forks become stalled or as damaged or missegregated chromosomes proceed through mitosis [72], [73], [74]. In some conditions, mammalian cells have been shown to adapt and proceed through the cell cycle in the presence of low levels of unrepaired double strand breaks [72], [75], [76], thus the weeks following exposure to radiation induced DNA damage could reproduce a similar state. Mechanisms promoting genomic instability include interference with DNA repair or increasing the damage burden. Our experiments showing IL-8 and senescence induction in response to increased DNA damage and the positive effects of the p38MAPK inhibitor, support the former possibility. Interference with DNA repair may be one of the mechanisms by which the secretory component of the p38MAPK-driven response could contribute to cell transformation and cancer progression [77].

The fact that most of the phenotypes observed in our studies are transient, leads us to speculate that cell proliferation is a factor contributing to the resolution of genomic instability either by leading to a dilution of the damage containing cell subpopulation or by an active positive influence. Consistent with this notion, animals exposed to protracted, low doses of ionizing radiation, show the accumulation of foci in non-replicating, but not in proliferating tissue such as the skin and intestine [78], [79]. Furthermore, non-proliferating fibroblasts, lung tissue, skin and mammary gland cells display persistent genomic instability for up to 24 months following exposure to ionizing radiation [79], [80], [81]. Repeated cycles of DNA synthesis could increase the opportunities for repair of persistently damaged DNA by homologous recombination and remodeling of the overall chromatin structure. Alternatively, the differential persistence over time of each phenotype could be explained by the disappearance of different cell populations. However, this model is difficult to reconcile with the high percentage of cells in the population positive for markers of proliferation, increased ROS levels, nuclear p38, intracellular IL-8 and IRIF at day 7 following exposure to radiation.

The stress response induced shares many phenotypes with senescence, which is a known outcome of exposure to high doses of radiation. Senescence is induced by multiple and varied initiators and executed by heterogeneous pathways [47], [54], [82]. Exposure to high doses of radiation (10–20Gy) triggers the activation of DDR which induces a feedback loop necessary and sufficient to establish an irreversible state of senescence characterized by growth arrest and sustained by p21, TGF-beta, p38MAPK activity, elevated ROS and DNA damage [83]. The contribution of this response to the biological effects of low and moderate doses of radiation remains unclear. At the dose (1Gy) primarily employed in this study, several of the markers shared with senescence where induced by high-LET radiation more effectively than low-LET radiation: p38MAPK, γH2AX foci, pro-inflammatory cytokine release and β galactosidase activity. However, the context for this response differed from established, irreversible senescence in several important aspects including: (i) occurs in proliferating cells, which by definition can not be senescent (ii) a lack of correlation of ROS levels with the amount of persisting genomic instability and with p38MAPK activity (iii) the transient nature of the stress-associated phenotypes, which disappeared at different times following radiation exposure and, (iv) the scarcity of cells in the population displaying more than 5 γH2AX foci. The later condition has been proposed by Passos and collaborators as a threshold required to establish the feedback loop leading to irreversible replicative senescence [83]. Rather, the observed response is more consistent with another condition termed stress induced premature senescence (SIPS), that may be present in proliferating cells, is driven mostly by p38MAPK activity and is independent of ROS status [84], [85].

A distinct response involving only some of the effectors and phenotypes involved in senescence could be the result of a weaker stimulus. Several responses associated with senescence, including replicative arrest [85], nuclear morphology alterations [86] and the secretory phenotype [48], have been shown to be induced by distinct mechanisms depending on the intensity of the stimuli. While high levels of p38MAPK activation are sufficient to induce these responses, at lower levels, this kinase cooperates with other effectors to promote senescence. The response induced by moderate radiation is similar to the phenotypes displayed by fibroblasts derived from various progeroid syndromes, where deficient activity of DNA damage management factors result in genomic instability associated with reduced population doublings over the cellular lifespan, however with proliferation in the context of phenotypes associated with senescence. Consistent with a threshold required to induce growth arrest, p38MAPK activity drives senescence in some of these syndromes while cooperatively modulates the response in others. For example, fibroblasts from Rothmund-Thompson (deficient in Req4 helicase activity) and from Seckel (ATR deficiency) syndrome patients exhibit replicative growth arrest that can be bypassed by telomerase expression, and p38MAPK dependent stress signaling only contributes to reduce the overall replicative capacity [87], [88]. In contrast, deficiencies in helicase Q in Werner Syndrome cells, leads to growth arrest that can be reversed by p38MAPK inhibition [89]. Thus, our results implicate p38MAPK activity in response to stress induced by high LET radiation, is sufficient to promote genomic instability, but insufficient to induce growth arrest.

Our results support a biological model illustrated in Figure 6 and, also suggest new strategies to mitigate radiation induced persistent responses as well as a specific effect for antioxidants as radioprotective versus radiation damage mitigating agents. We propose that cellular proliferation, as well as p38MAPK driven responses, are factors regulating the persistence of radiation exposure-induced phenotypes. Our model is also consistent with the observed extended persistence of genomic instability in non-proliferating cell types and tissues, and implicates tissue-specific factors governing the robustness of these responses induced by low to moderate levels of radiation. Our studies also have implications for translational applications for radiation exposures in humans as we speculate that two potential interventions to reduce persistent genomic instability would be to alter the cellular redox balance towards a moderately pro-oxidative metabolism to elicit homeostatic pathways, as well as employing measures to counteract p38MAPK-signaling pathways.

## Supporting Information

Figure S1 **A Histograms of 10,0000 HBEC-3KT cells analyzed for DCF fluorescence at day 7 following exposure to sham irradiation (black line), 1Gy X-rays (blue line) or 1Gy Fe ions (red line).** Although the total increase in fluorescence is small, the complete population shifts toward higher fluorescence levels. **B** Dot plot of DCF fluorescence vs. DNA content using 5 µM Draq 5 in 5,000 cells at day 5 following exposure to sham irradiation (black line), or 1Gy X-rays (blue line). This experiment shows that cells in G1 as well as in G2 phases of the cell cycle increase the average DCF fluorescence following irradiation. **C** Clonogenic survival assay of HBEC-3KT cells following exposure to low-LET radiation. HBEC-3KT cells were plated at a density of 200 cells per well, exposed to the indicated X-ray dose 14 h later and cultured for 15 days. Colonies with more than 50 cells were counted. 1 of 2 experiments is shown.
(TIF)

Figure S2 **A Control for SA beta galactosidase staining.** Primary human foreskin fibroblasts (p3) were stained for beta galactosidase at day 7 following exposure to 10Gy X-rays. In contrast to HBEC-3KT cells, positively stained cells can be clearly distinguished among non stained cells (arrows). **B** HBEC-3KT cells display increased nuclear area at day 7 following exposure to 1Gy Fe ions (green traces) but not to 1Gy X-rays (blue traces) compared to non irradiated cells (black traces). The pixel area of 200 nuclei for each condition were measured using Image J. Frequency distribution graphs were generated by binning the data. The arrows indicate the mean of the population. Assuming distributions of similar shape and close enough to normal, one way ANOVA: Non IR 1 or 2 vs. Fe1, Fe2, Fe3 p<0.0001. Non IR1 or IR2 vs. X1, X2 or X3 p = 1. X1, X2, X3 vs F1, F2 or F3: p<0.05. Independent irradiation replicates are represented in different color shades.
(TIF)

Figure S3 **Low LET radiation (X-rays) induces a senescence-like response in cycling cells. A** IL-8 detected by ELISA in conditioned media 7 days following exposure to the indicated X-rays dose. **B** Immunofluorescence detection of 53BP1 positive foci, p38MAPK and intracellular IL-8 in cells treated with 3 µg/ml cytochalasin B for 6 h prior to fixation at day 7 following exposure to 6Gy X-rays. To detect IL-8, secreted proteins were accumulated in intracellular compartments by treatment with pH gradient disrupting drugs such as Monensin (2 µM) or Brefeldin A (10 µg/ml) for 90 minutes before fixation. To detect this antigen, the cells were permeabilized with 0.1%saponin. **C** Control experiment to demonstrate that p38MAPK becomes phosphorylated and translocates to the nucleus upon treatment with stressors such as 1 h incubation with 10 µM anisomycin. Naive cells do not have nuclear p38 or phosphorylated p38MAPK.
(TIF)

Figure S4 **Western Blot analysis of cell lysates prepared from scramble, ATM or p38MAPKα siRNA transfected cells.** Western blot shows efficient interference with protein expression 4 days after the first transfection and that ATM is not an off-target of p38MAPKα knock-down and vice versa. Antibodies used: ATM (GeneTex, Irvine CA, USA), actin (Sigma).
(TIF)

Figure S5 **A Immunofluorescence staining for γH2AX and 53BP1foci to exclude interference of H_2_O_2_ treatment with DNA repair foci detection.** Cells shown in panel A were exposed to 3Gy X-rays and seven days later treated with 10 µM H_2_O_2_ in PBS (H_2_O_2_) or PBS alone (Control) for 15 minutes on ice prior to fixation with 4% PFA and staining to demonstrate that H_2_O_2_ treatment does not interfere with antigen detection by immunoflurescence. Scale bar = 10 µm. **B**, DCF formation measured by flow cytometry in response to increasing dose of H_2_O_2_ added to labeled HBEC-3KT cells.
(TIF)
