# Supplementary figures and images for: Opposite Roles for p38MAPK-Driven Responses and Reactive Oxygen Species in the Persistence and Resolution of Radiation-Induced Genomic Instability
Source: PLoS One. 2014 Oct 1;9(10):e108234. doi: 10.1371/journal.pone.0108234 (PMC4182705; doi:10.1371/journal.pone.0108234)

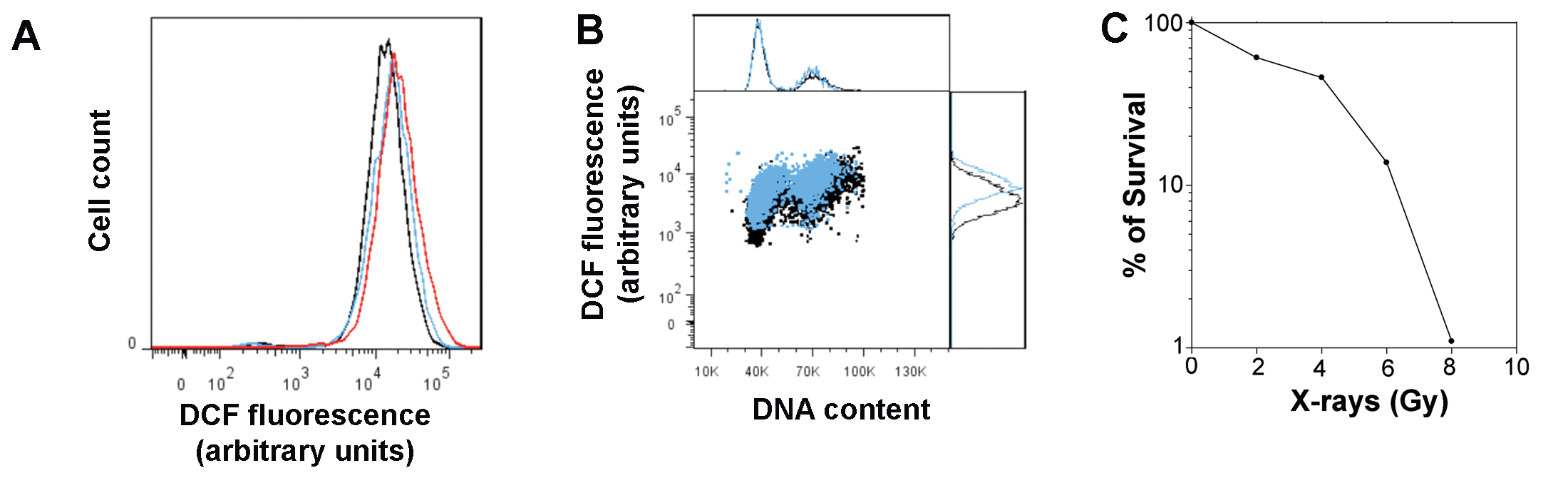

Supplement: Figure S1 — A Histograms of 10,0000 HBEC-3KT cells analyzed for DCF fluorescence at day 7 following exposure to sham irradiation (black line), 1Gy X-rays (blue line) or 1Gy Fe ions (red line). Although the total increase in fluorescence is small, the complete population shifts toward higher fluorescence levels. B Dot plot of DCF fluorescence vs. DNA content using 5 µM Draq 5 in 5,000 cells at day 5 following exposure to sham irradiation (black line), or 1Gy X-rays (blue line). This experiment shows that cells in G1 as well as in G2 phases of the cell cycle increase the average DCF fluorescence following irradiation. C Clonogenic survival assay of HBEC-3KT cells following exposure to low-LET radiation. HBEC-3KT cells were plated at a density of 200 cells per well, exposed to the indicated X-ray dose 14 h later and cultured for 15 days. Colonies with more than 50 cells were counted. 1 of 2 experiments is shown. (TIF) [file pone.0108234.s001.tif]

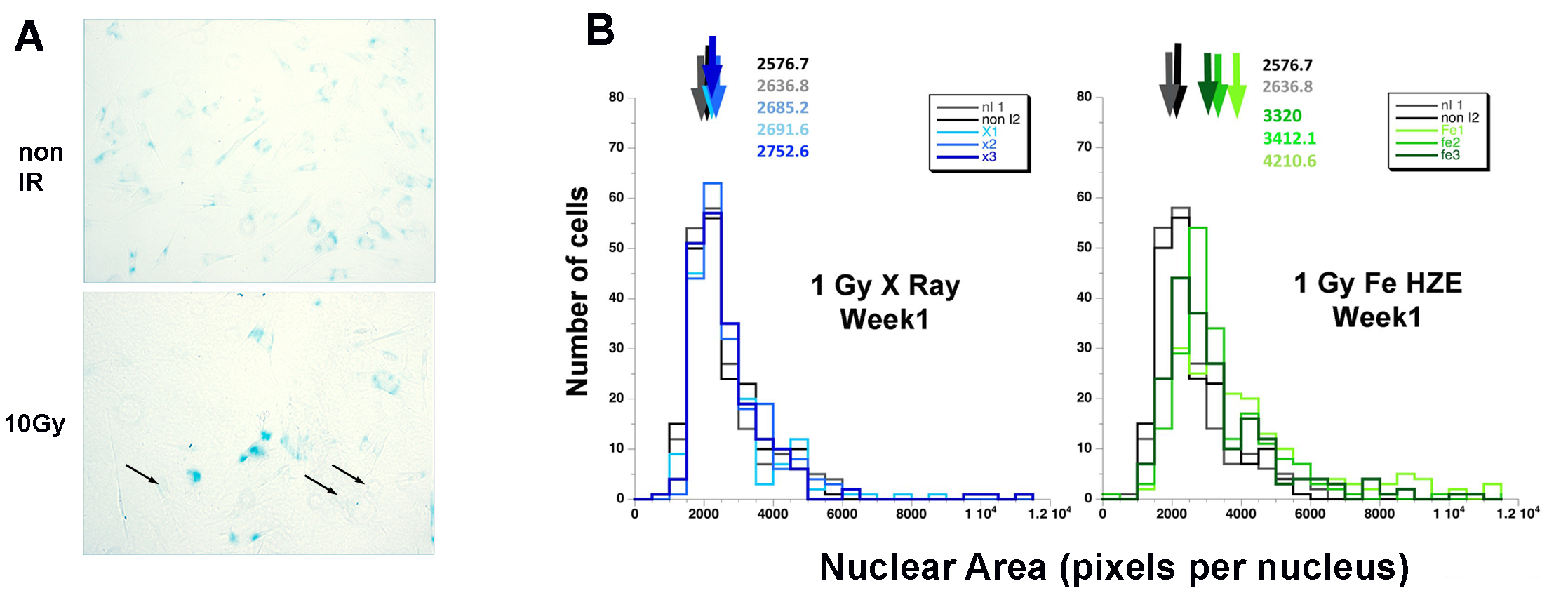

Supplement: Figure S2 — A Control for SA beta galactosidase staining. Primary human foreskin fibroblasts (p3) were stained for beta galactosidase at day 7 following exposure to 10Gy X-rays. In contrast to HBEC-3KT cells, positively stained cells can be clearly distinguished among non stained cells (arrows). B HBEC-3KT cells display increased nuclear area at day 7 following exposure to 1Gy Fe ions (green traces) but not to 1Gy X-rays (blue traces) compared to non irradiated cells (black traces). The pixel area of 200 nuclei for each condition were measured using Image J. Frequency distribution graphs were generated by binning the data. The arrows indicate the mean of the population. Assuming distributions of similar shape and close enough to normal, one way ANOVA: Non IR 1 or 2 vs. Fe1, Fe2, Fe3 p<0.0001. Non IR1 or IR2 vs. X1, X2 or X3 p = 1. X1, X2, X3 vs F1, F2 or F3: p<0.05. Independent irradiation replicates are represented in different color shades. (TIF) [file pone.0108234.s002.tif]

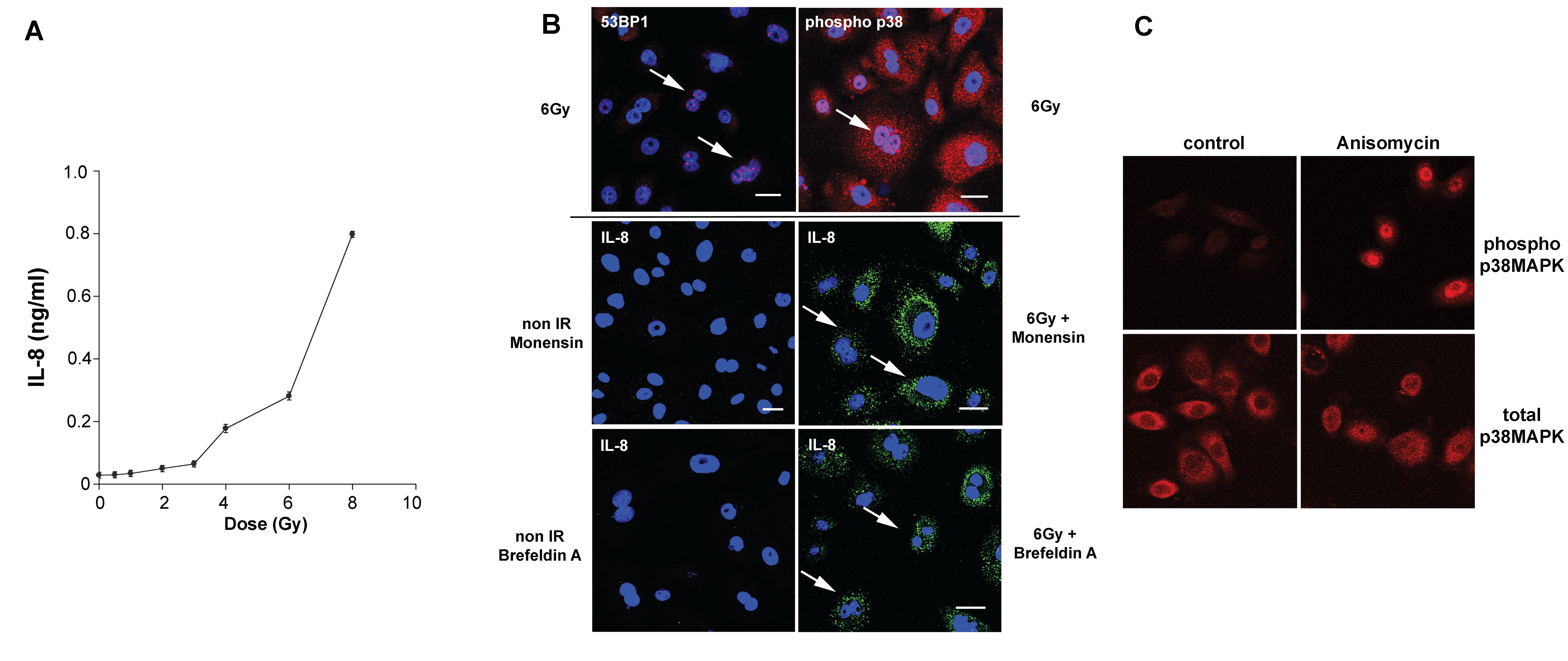

Supplement: Figure S3 — Low LET radiation (X-rays) induces a senescence-like response in cycling cells. A IL-8 detected by ELISA in conditioned media 7 days following exposure to the indicated X-rays dose. B Immunofluorescence detection of 53BP1 positive foci, p38MAPK and intracellular IL-8 in cells treated with 3 µg/ml cytochalasin B for 6 h prior to fixation at day 7 following exposure to 6Gy X-rays. To detect IL-8, secreted proteins were accumulated in intracellular compartments by treatment with pH gradient disrupting drugs such as Monensin (2 µM) or Brefeldin A (10 µg/ml) for 90 minutes before fixation. To detect this antigen, the cells were permeabilized with 0.1%saponin. C Control experiment to demonstrate that p38MAPK becomes phosphorylated and translocates to the nucleus upon treatment with stressors such as 1 h incubation with 10 µM anisomycin. Naive cells do not have nuclear p38 or phosphorylated p38MAPK. (TIF) [file pone.0108234.s003.tif]

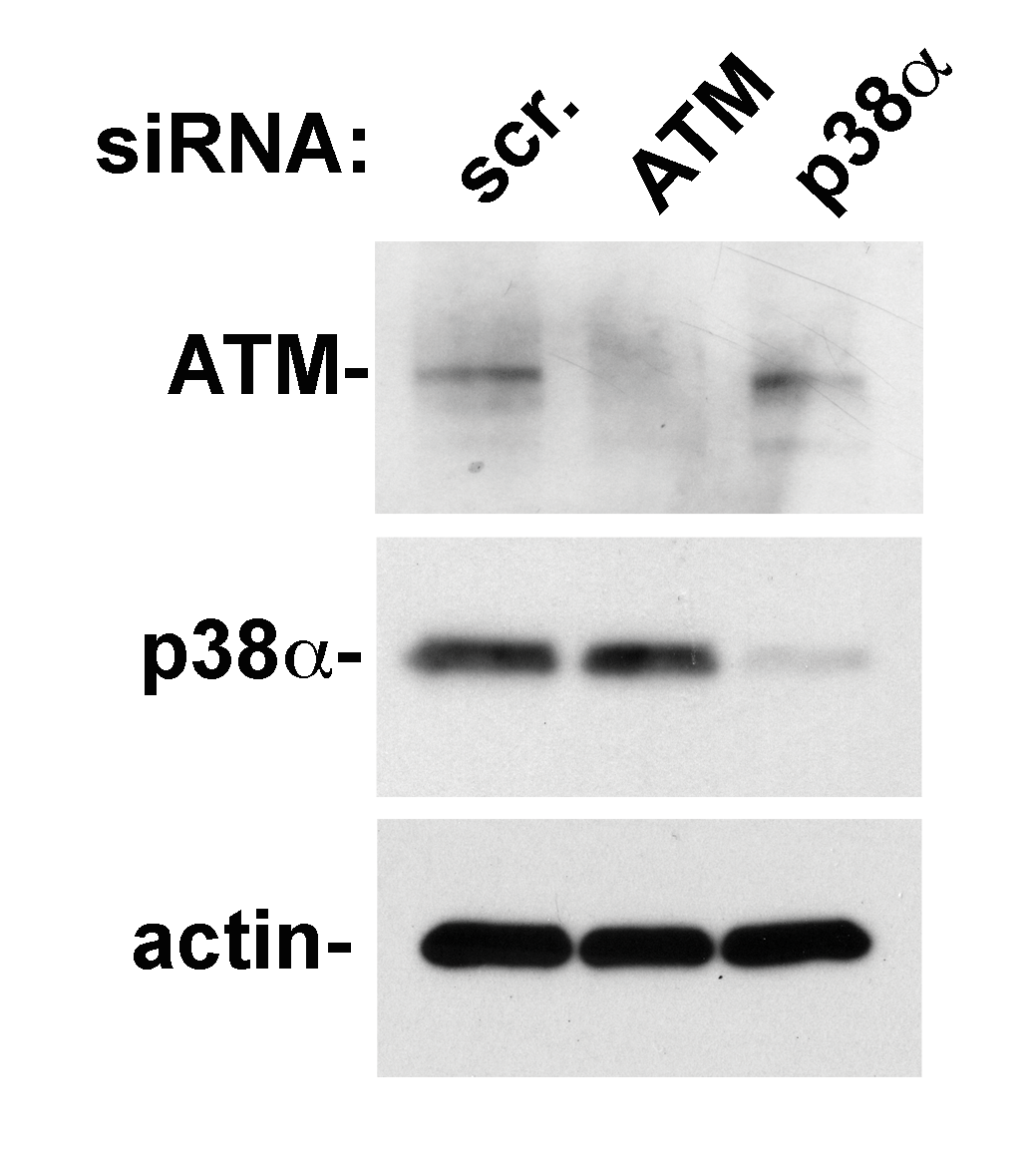

Supplement: Figure S4 — Western Blot analysis of cell lysates prepared from scramble, ATM or p38MAPKα siRNA transfected cells. Western blot shows efficient interference with protein expression 4 days after the first transfection and that ATM is not an off-target of p38MAPKα knock-down and vice versa. Antibodies used: ATM (GeneTex, Irvine CA, USA), actin (Sigma). (TIF) [file pone.0108234.s004.tif]

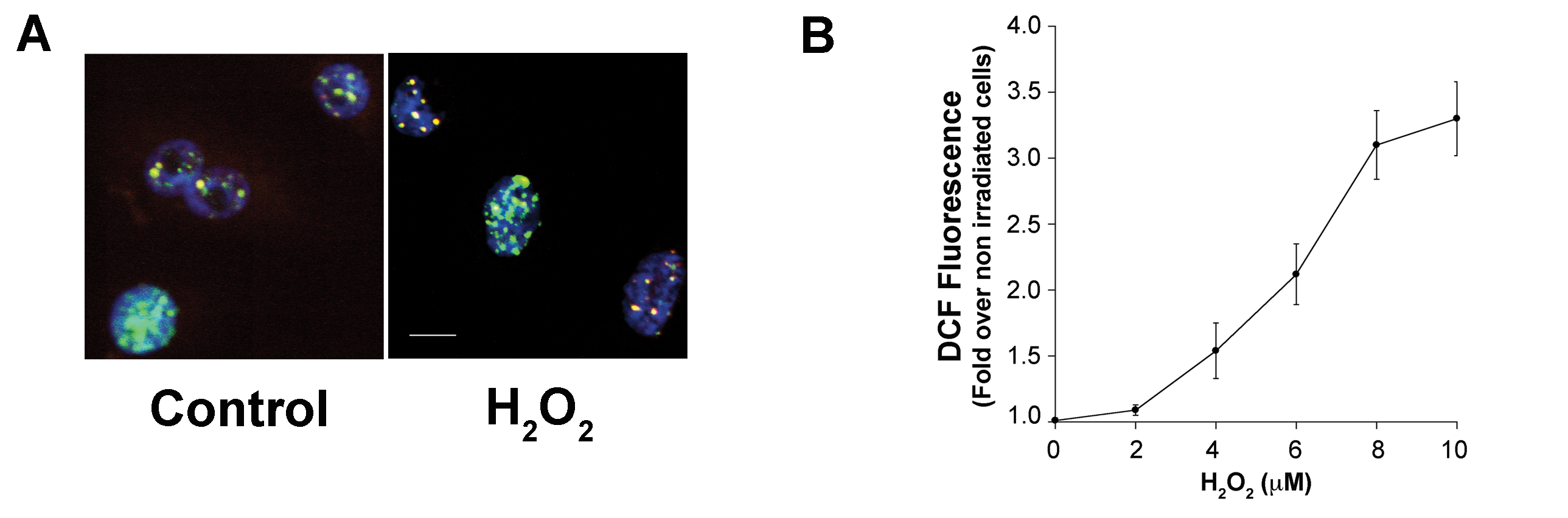

Supplement: Figure S5 — A Immunofluorescence staining for γH2AX and 53BP1foci to exclude interference of H2O2 treatment with DNA repair foci detection. Cells shown in panel A were exposed to 3Gy X-rays and seven days later treated with 10 µM H2O2 in PBS (H2O2) or PBS alone (Control) for 15 minutes on ice prior to fixation with 4% PFA and staining to demonstrate that H2O2 treatment does not interfere with antigen detection by immunoflurescence. Scale bar = 10 µm. B, DCF formation measured by flow cytometry in response to increasing dose of H2O2 added to labeled HBEC-3KT cells. (TIF) [file pone.0108234.s005.tif]
